# Spray on skin for diabetic foot ulcers: an open label randomised controlled trial

**DOI:** 10.1186/s13047-019-0362-x

**Published:** 2019-11-15

**Authors:** Laurens Manning, Emma J. Hamilton, Edward Raby, Paul E. Norman, Wendy Davis, Fiona Wood, Keryln Carville, Mendel Baba, Jonathan Hiew, Erica Ryan, Ivana Ferreira, Paul Gittings, Jens C. Ritter

**Affiliations:** 1 Department of Infectious Diseases, Fiona Stanley Hospital, 11 Robin Warren Drive, Murdoch, WA 6150 Australia; 2 Endocrinology Department, Fiona Stanley Hospital, 11 Robin Warren Drive, Murdoch, WA 6150 Australia; 3 Medical School, University of Western Australia, Crawley, WA 6009 Australia; 4 Department of Vascular Surgery, Fremantle Hospital, Alma Street, Fremantle, WA 6160 Australia; 5 State Burns Unit, Fiona Stanley Hospital, 11 Robin Warren Drive, Murdoch, WA 6150 Australia; 6 Silver Chain Group and School of Nursing and Midwifery, Curtin University, Bentley, WA 6102 Australia; 7 Podiatry Department, Fiona Stanley Hospital, 11 Robin Warren Drive, Murdoch, WA 6150 Australia; 8 Burns Department, Fiona Stanley Hospital, 11 Robin Warren Drive, Murdoch, WA 6150 Australia; 9 Department of Vascular Surgery, Fiona Stanley Hospital, 11 Robin Warren Drive, Murdoch, WA 6150 Australia

**Keywords:** Diabetic foot ulcer, Wound healing, ReCell®, Autologous skin cell harvest

## Abstract

**Background:**

One Australian loses a limb every 3 h as a result of infected diabetic foot ulcers (DFU). This common condition accounts for substantial morbidity and mortality for affected individuals and heavy economic costs for the health sector and the community. There is an urgent need to test interventions that improve wound healing time, prevent amputations and recurrent ulceration in patients presenting with DFU whilst improving quality of life and reducing health care costs.

**Methods:**

One hundred and fifty eligible participants will be randomised to receive an autologous skin cell suspension, also termed ‘spray-on’ skin (ReCell®) or standard care interventions for their DFU. The primary outcome is complete wound healing at 6 months, but participants will be followed up for a total of 12 months to enable secondary outcomes including total overall costs, ulcer free days at 12 months and quality of life to be assessed.

**Discussion:**

Outpatient costs for dressings, home nursing visits and outpatient appointments are key cost drivers for DFU. If spray-on skin is effective, large cost savings to WA Health will be realised immediately through a shortened time to healing, and through a higher proportion of patients achieving complete healing. Shortened healing times may enable participants to return to work earlier. Any economic benefits are likely to be amplified across Australia and other similar demographic settings where aging populations with increased diabetes rates are considered major future challenges.

**Trial registration:**

Australian New Zealand Clinical Trials Registry ACTRN12618000511235. Registered on 9 April 2018.

## Background

One Australian loses a limb every 3 h as a direct consequence of diabetes related foot disease, usually due to an infected diabetic foot ulcer (DFU) [1, 2]. That amounts to around 8000 lower extremity amputations (LEA) undertaken in Australia each year [3, 4]. In Western Australia (WA) alone, more than 200 major (above the ankle) LEA are performed annually and recurrent minor (below the ankle) amputations in patients with Type 2 diabetes (T2DM) are increasing by about 3.5% per annum [5]. There has been a 30% increase in diabetes-related amputations, largely related to increasing diabetes prevalence [6] in Australia over the past decade and 8% of diabetes-related deaths are attributable to foot complications [1, 2, 7]. These poor outcomes have persisted despite increasing awareness of the medical, economic and social burden of diabetic foot complications [8]. The economic costs and mortality rates exceed that of many common cancers; the 5-year mortality rate of patients with diabetic foot infections is ~ 50% [9–12]. The estimated economic burden in Australia may exceed $1.5 billion, with DFU accounting for 33% of diabetes related costs [13]. Poor outcomes associated with DFU are disproportionately high in the Australian Indigenous population [14]. A recently published systematic review has found that Aboriginal and Torres Strait Islander Australians are 3–6 times more likely to experience a diabetes related foot complication than non-Aboriginal Australians [15]. Therefore there is an urgent need to test interventions to improve healing time, reduce recurrent ulceration and the incidence of LEA in patients with DFU whilst optimising function and quality of life.

In addition to in-patient services required when the DFU becomes limb or life threatening, most of the morbidity and economic burden of DFU is carried by public sector outpatient services. Wound healing time is a key cost driver and influences the overall cost-benefit analysis for any DFU intervention [16]. It is estimated that the mean healing time for a DFU managed without amputation is 6 months, rising to 12 months if an amputation is required [17, 18]. The outpatient component of management accounts for 71% of the total costs associated with DFU and suggests that reductions in the time to healing are likely to have major benefits for direct costs, particularly related to home nursing visits, dressings and outpatient appointments.

### Rationale

We hypothesise that the use of ‘spray-on’ autologous skin grafting (ReCell®; Avita Medical) in DFU will decrease healing time and thereby reduce overall cost of treatment. Autologous ‘spray on’ skin aids epithelial regeneration and has been used successfully in the treatment of scars and burns and other ulcers [19], particularly when traditional split skin grafting is not feasible. Although it has shown some early promise in a small case series of 4 ft ulcers [20] and for other chronic ulcers [19], no randomised trial of this product has been completed or is currently planned. The aim of this study is to assess the potential benefit of spray-on skin as a superior, and cost-effective, management strategy for DFU.

### Hypotheses

1. Spray-on autologous skin grafting improves wound healing time in patients with DFU compared with standard care
2. Spray-on autologous skin grafting is cost-effective when compared with standard care

## Study design

This study is a prospective, randomised, open label trial powered for superiority. Local and international data informed sample size calculations indicating that, at present, 45% of patients with DFU will achieve complete healing at 6 months [17, 18, 21]. We estimate that 136 (with continuity correction) patients are required to have 80% chance of detecting, at the 5% level of significance, an increase in the primary outcome measure from 45% in the control group to 70% in the spray-on skin group [19]. To account for drop-outs, we will aim to recruit 150 participants in total.

### Study population

The study population will be screened from patients attending the Fiona Stanley (FSH) or Royal Perth Hospital (RPH) inpatient or outpatient multidisciplinary foot units during the recruitment period. The primary ulcer requiring treatment will be considered the index ulcer for enrolment. The site of the index ulcer will be defined at enrolment and categorised as either being fore-, mid- or hindfoot. During the lead-in phase, the wound bed preparation will be standardised prior to randomisation. Recruitment will occur over an 18-month period and participants followed for 12 months.

### Randomisation

Randomisation will take place 2 weeks (+/− 6 days) from the last significant debridement or minor amputation to allow the surgical wound site to demonstrate early healing. Randomisation into treatment (ReCell®) or control (standard care) is performed by randomisation program (REDCap) and researchers are blinded to the randomisation algorithm that will include variable block sizes that are randomly 2, 4 or 8.

### Inclusion criteria

i) Age ≥ 18 years
ii) Diabetes (type 1 or 2) defined according to international consensus guidelines
iii) Admission to FSH or RPH, or visit to outpatients departments with a DFU requiring local debridement or minor amputation
iv) Ulcer area > 6 cm^2^
v) The ulcer location, contour, shape and wound base is deemed to be suitable for administration of spray on skin
vi) No further debridement or amputation is anticipated
vii) Wound bed is adequately vascularised as determined by the presence of at least one palpable pulse in the affected foot, or at least single vessel run off identified by arterial Doppler ultrasonography, MRI, CT or conventional angiography (including following revascularisation procedures)
viii) Competent and willing to provide informed consent
ix) Able to be followed up by ambulatory care services (Silver Chain) for community nursing

### Exclusion criteria

i) Non-diabetic ulcer
ii) Wounds deemed unsuitable on the basis of contour, location, vascularity or other factors
iii) Limb threatening ischaemia or sepsis requiring early major amputation
iv) Not competent to provide informed consent
v) Unlikely to be accessible for follow-up visit over the next 12 months

### Primary outcome

The primary outcome for the trial will be a dichotomous outcome of complete healing of the index ulcer at 6 months as defined by full epithelialisation, after debridement of callus, lasting for at least 2 weeks. Primary outcome arbitration at the interim analysis and at the final analysis will be performed using the database, wound dimension and clinical images assessed by two independent senior clinicians (not investigators) blinded to the intervention. Discordant outcome assessments will be resolved by consensus.

### Secondary outcomes

Secondary outcomes include: i) index ulcer free days at 12 months, ii) time to full epithelialisation of the index ulcer, iii) trajectory of wound healing of the index ulcer (defined as volume and measured using Silhouette™), iv) major adverse events, v) any minor or major lower limb amputation, vi) all-cause mortality, vii) re-ulceration of the index ulcer, viii) the development of any new ulcers ix) total costs of inpatient and outpatient costs (see economic analysis, below), x) readmission to hospital and xi) health-related quality of life (as measured by EQ-5D-5 L) [22].

The definitions for secondary outcomes are consistent with international guidelines [23]. Re-ulceration is defined as healing of index ulcer followed by subsequent ulceration with loss of epithelialisation at the same location. A minor amputation is an amputation below the ankle including toe, metatarsal-phalangeal and midfoot amputations. A major amputation is an amputation above the ankle including below knee and above knee amputations. New onset infection includes infections that have commenced during the preceding 2 weeks, including existing ulcers that have never previously been infected.

## Methods

### Pre-intervention – wound bed preparation

To optimise the quality of the wound bed prior to administration of autologous spray-on skin, wound bed preparation will be standardised; all patients identified for inclusion into the trial will be receive the same pre-intervention protocol before randomisation. For wounds > 1 cm deep, negative pressure dressings will be applied with a Prontosan® [B. Braun] soak at each change. For wounds < 1 cm deep, IntraSite conformable [Smith & Nephew] (with Prontosan® soak as above) will be used.

On the day of randomisation, the index wound will be further cleansed via ultrasonic debridement to remove as much biofilm and devitalised tissue as possible. This will allow for uniformity in wound base appearance prior to randomisation. After ultrasonic debridement, wounds will be swabbed and the swabs stored for future microbiome analysis.

### Intervention – spray on skin

Through a series of validated steps, ReCell® [Avita Medical] enables disaggregation of cells from a patient’s skin and the preparation of a suspension of these cells that can be sprayed or dropped directly onto the prepared wound bed.

The skin from which the suspension is prepared comes from a small split skin graft (SSG) collected from the patients’ upper thigh. An electric dermatome set at 2 mm depth or a scalpel blade (size 10) placed at a shallow angle will be used to harvest the skin. In comparison with traditional SSG, the size of the sample for the spray on skin preparation is much smaller and will be approximately 2cm^2^. The harvest site area will be recorded to control for the dose of skin cells applied as a possible co-variate in statistical analyses.

A small amount of the harvested skin preparation will be reapplied to the harvest site to enable more rapid healing. The harvest site will be dressed with Surfasoft® [Tauren] and Mepilex® Border [Mölnlycke].

The process of disaggregating the cells is performed with ReCell® [Avita Medical] as previously described in [20] and relies on the enzyme trypsin to allow the epidermis to be separated from the dermis. The cells at the epidermal-dermal junction can then be scraped off using a scalpel and are collected and filtered before being dropped or sprayed onto the wound site. Immediately after autologous skin application the index wound will be dressed with Surfasoft® dressing for a minimum of 5 days. Secondary absorbent dressings may be changed as required without disturbing the Surfasoft® layer during this time to allow adequate cell adherence.

Patients randomised into the control arm will continue with standard wound care procedures as per normal day to day proceedings of the hospital Outpatients clinic following ultrasonic debridement.

### Follow up measurements

Patients will be followed up at predefined time points. This will coincide with routine outpatient visits ordinarily scheduled at 4, 10, 18, 26, 39 and 52 weeks from randomisation with phone consultations every fortnight to determine total ‘ulcer-free’ days following healing. At each scheduled visit, the wound will be assessed in terms of location (exact; forefoot vs. hind- and mid-foot), depth, dimensions, wound volume and wound area (as measured by Silhouette™ [Aranz Medical]), wound quality (patchy vs. confluent, % epithelialisation), probe to bone test and photography. Both feet will be assessed for new wounds. Blood tests will be (lipids, C-reactive protein, full blood examination and renal and liver function tests) will be performed as clinically indicated, but an HbA_1c_ will be measured at 3, 6 and 12 months to determine the change from recruitment. All patients will receive standard care related to off-loading, ongoing diabetes management and infection management as per the usual management protocol of the multi-disciplinary foot ulcer teams. In addition to baseline, an EQ-5D-5 L [22] will be performed at 26 and 52 week visits to ascertain change in health-related quality of life [24]. The primary outcome will be assessed at the 26-week visit.

### Adverse events

Although it is expected that the intervention will be safe, adverse events (AE) will be pre-specified and reported to the Data Safety and Monitoring Board (DSMB; see below) in accordance with NHMRC position statement of monitoring and reporting of clinical trials. A DSMB will be established that includes an independent researcher and two independent clinicians including one with experience in clinical trials and the other with managing diabetic foot infections. As this is not a systemic intervention, there will be no stopping rules based on any haematological or biochemical parameters.

A severe adverse event (SAE) form will be reported promptly to the DSMB if any of the following occurs:

1. Death from any cause
2. Major limb amputation of the same leg as the index ulcer at any stage up to 12 months from enrolment
3. Major infection of the harvest site as defined as the requirement for admission to hospital, surgical debridement or intravenous antibiotics

An AE form will be reported if any of the following occurs:

1. Readmission for any reason related to infection or deterioration of the index ulcer
2. Minor amputation unrelated to the index ulcer, but on the same foot as the index ulcer (after enrolment)
3. Minor infection of the harvest site as defined by erythema and the requirement for oral antibiotic therapy for this
4. Delayed healing of the harvest site as defined by persistent need for a dressing on the harvest site at or beyond the 4 week visit

## Statistical considerations

### Primary and secondary outcomes

All analyses will be conducted according to the intention-to-treat principle. Baseline characteristics will be compared by treatment group. Effects of treatment on the primary study endpoint (complete healing of the index ulcer at 6 months) will be estimated with the use of unadjusted logistic regression with last observation carried forward for those lost to follow-up. All *P*-values will be two-sided and *P*-values less than 0.05 will be considered to indicate statistical significance. Multiple logistic regression will be applied to adjust for prognostic factors such as vascular insufficiency and site of DFU (fore-, mid- or hind foot) which are strongly correlated with the outcome (*r* ≥ 0.3) [25, 26]. Binary secondary outcomes at 12 months will be analysed similarly. For continuous secondary outcomes, change score analysis that determines treatment effect based on the difference between baseline and post treatment score (basic adjustment) will be undertaken. Multiple linear regression adjusting for i) the baseline value of the outcome variable in the model (model 1), and ii) model 1 + adjustment for prognostic baseline factors (model 2) will be undertaken. Data will be analysed using IBM SPSS Statistics 25.

### Subgroup analyses

Pre-specified sub-group analyses will be performed using the dichotomous primary study endpoint described above. Sub-groups include ulcer site (categorical variable; fore-, mid- or hind foot, plantar/dorsal), WiFI Clinical Stage [27] at baseline presentation (categorical/ordinal variable; clinical stage 1–4), co-existent moderate to severe renal disease (dichotomous variable; creatinine clearance ≤30 mL/min, age (dichotomous variable; age ≤ 60 years) long term diabetic control at presentation (dichotomous variable; HbA1C ≤9%), primary surgical procedure performed (dichotomous variable; minor amputation vs. local sharp or surgical debridement).

### Interim analysis

Due to the long-time delay until the primary outcome can be ascertained for each patient, we plan a single interim analysis after the first 80 patients. We estimate that 78 (with continuity correction) patients are required to have 80% chance of detecting, as significant at the 1% level, an increase in the primary outcome measure from 45% in the control group to 85% in the spray-on skin group [19]. If this threshold is met, the trial will be ceased early. If this threshold is not met, the trial will be completed as described above.

### Health economic analyses

The main perspective of the analyses will be societal. Direct health care costs including spray on skin and usual care treatment costs, hospital inpatient and outpatient costs, including hospital in the home, out-of-hospital medical services and consumables will be estimated. An incremental cost-effectiveness analysis will be performed in which the net costs and net effectiveness of spray on skin will be compared with those of usual care and expressed as ratios. All analyses and comparisons will be performed on an intention-to-treat basis. Since the time horizon for the primary endpoint (complete healing of the index ulcer) is 6 months, discounting will not be performed. The confidence intervals for the incremental cost-effectiveness ratio will be estimated using the bootstrap approach with 1000 repeated random samples drawn with replacement from the original data. Bootstrap confidence intervals will be constructed with the bias-corrected percentile method. Data will be analysed using IBM SPSS Statistics 25 [IBM Corporation].

Overall costs will be determined by length of hospital stay, number and nature of operating theatre visits, use of pathology and radiology services, length of “hospital in the home” treatment, direct antibiotic costs, consumables associated with wound management (dressings, debridement, human skin replacements, negative pressure wound therapy dressings; ambulatory nursing attendance, orthotic appliances (prostheses, casting, shoes, in-soles). To ascertain data on direct costs for outpatient home care nursing we will extract data from the ambulatory nursing services electronic data collection tool (COMCARE™ [Docobo]). Outpatient visit costs will be derived from Medicare rebates (for multidisciplinary team visits and single specialty attendances at podiatry, infectious diseases or vascular clinics), whilst drug costs will be estimated from listed drug costs from Pharmaceutical Benefits Scheme (PBS) published listing, or in the case of moxifloxacin, piperacillin-tazobactam, ertapenem and other non-PBS drugs, from direct pharmacy costs. All costs will be adjusted on a year-by-year basis according to the medical services component of the Consumer Price Index to a single financial year for comparisons (2018).

### Data management procedures

Study data will be collected and managed using REDCap [9.2.5 Vanderbilt University] electronic data capture tools hosted at the University of Western Australia [28].

## Discussion

There are substantial barriers to undertaking randomised controlled trials in patients with DFU. Significant heterogeneity in lower limb vascular supply, ulcer location and size, long-term diabetes control, infection extent, antibiotic efficacy, adherence to offloading and psychosocial issues are all factors that affect healing and may compromise the validity of testing a novel intervention to improve cure rates in this group. Restricting entry criteria may reduce heterogeneity in the trial participants, but places limits on generalisability of the results to other patients outside this group. For example, excluding patients with established osteomyelitis in the ‘SIDESTEP’ trial comparing ertapenem with piperacillin-tazobactam for diabetic foot disease limited generalisability to patients with this common complication [29].

In the case of the spray on skin intervention for the present trial, the goal was to maximise generalisability to as many patients with a DFU at ‘moderate’ risk of delayed healing. Because the additional costs are likely to be sensitive to the cost of ReCell®, smaller wounds with a high likelihood of healing, were not a suitable application for this intervention. Such wounds would include a transphalangeal amputation site in a patient with good blood supply. Likewise, limiting the intervention to a salvage therapy for wounds with an extremely high chance of clinical failure would also compromise the chances of demonstrating success.

Finding the balance between patient heterogeneity, prior knowledge about likelihood of success and the subsequent generalisability of the trial results is difficult and may have implications for the appropriate sample size calculations. In this trial, healing rates greater than 45% in the standard care arm from published data may not necessarily reflect outcomes for this type of ulcer in our tertiary multidisciplinary unit.

That slow recruitment rate is a challenge to this trial is not unexpected. Studies have demonstrated that only a third of well-funded trials manage to maintain planned recruitment schedules [30]. For the present study, the commonest reason for patients with DFU that would be ordinarily be suitable for the trial is accessibility. Western Australia covers a large area, and our catchment covers patients from distances greater than 500 km. Our experience thus far is that patients > 60 min travel away may struggle to access ambulatory care services and together with regular travel to FSH precludes recruitment. Our approach to improve patient recruitment was to expand to include RPH as a second site. By replicating the trial infrastructure in another Perth metropolitan tertiary hospital, we hope to improve the catchment of patients. Refusal of eligible patients to participate in this trial has been reassuringly uncommon.

## Data Availability

Not applicable. We have not yet generated any data as part of this trial.

## Abbreviations

AE: adverse event
CT: computer tomography
DFU: diabetic foot ulcer
EQ-5D-5 L: European QoL − 5 dimensions survey
FSH: Fiona Stanley Hospital
HbA1c: glycated haemoglobin
HREC: health research ethics committee
LEA: lower extremity amputation
LoS: length of stay
MRI: magnetic resonance imaging
PBS: pharmaceutical benefits scheme
QoL: Quality of life
REDCap: Research Electronic Data Capture
RPH: Royal Perth Hospital
SAE: severe adverse event
SMHS: South Metropolitan Health Service
SSG: split skin graft
T2DM: Type 2 diabetes mellitus
WA: Western Australia
